# Relationship between nerve injury-induced protein gene 2 polymorphism and stroke in Chinese Han population^☆^

**DOI:** 10.1016/S1674-8301(11)60039-0

**Published:** 2011-07

**Authors:** Xin Wang, Jianying Zhang, Yi Liu, Yingdong Zhang

**Affiliations:** a Department of Neurology, Medical School of Nanjing Medical University, Nanjing, Jiangsu 210029, China;; b Department of Neurology, Nanjing Brain Hospital, Nanjing, Jiangsu 210029, China.

**Keywords:** nerve injury-induced protein 2 (NINJ2), single nucleotide polymorphism (SNP), stroke, cell adhesion molecule

## Abstract

The aim of present study was to investigate the relationship between nerve injury-induced protein 2 (*NINJ2*) gene polymorphism and stroke in Chinese Han population. Fifty-two patients with large-artery atherosclerosis (LAA) infarction, 85 patients with small-artery occlusion lacunar (SAO) infarction, 50 patients with intracerebral hemorrhage (ICH) and 66 controls were included. Genotypes and alleles frequencies of the two single nucleotide polymorphisms (SNPs) of *NINJ2* among different groups were analyzed and compared. In regard to *rs12425791*, the frequencies of the *AG* and *AA*+*AG* genotypes of the LAA and SAO groups were significantly higher than those in the control group; the frequency of the *A* allele of the SAO group was significantly higher than that of the control group. In regard to *rs11833579*, there were not any significant differences between the case and the control groups. The SNP *rs12425791* is significantly associated with ischemic stroke, and the *A* allele increases the susceptibility to stroke. The SNP *rs11833579* is not significantly associated with stroke.

## INTRODUCTION

Stroke is a disease with high prevalence, and is especially notable for its high mortality and disability rate, inflicting a heavy socioeconomic burden. Many risk factors, such as hypertension, diabetes, hyperlipidemia, smoking and heart disease have been identified nowadays, but the genetic background of stroke still remains unclear.

A prospective cohort study found that two single nucleotide polymorphisms (SNPs) of the nerve injury-induced protein 2 (*NINJ2*) gene were significantly associated with stroke, and the two minor alleles of this gene increased the susceptibility to stroke, especially to the atherosclerotic thrombotic subtype[1]. The two SNPs were close to the 5′ end of the *NINJ2* gene, indicating that *NINJ2* may play an important role in stroke. But the study was only carried out in the Caucasian and Black population, while the relationship between the two SNPs and stroke in other populations is still unknown. Heve, we carried out a case-control study to investigate the relationship between the two SNPs and stroke in Chinese Han population. Fifty-two patients with large-artery atherosclerosis (LAA) infarction, 85 patients with small-artery occlusion lacunar (SAO) infarction, 50 patients with intracerebral hemorrhage (ICH) and 66 controls were included in this study. Genotypes of the two SNP sites among different groups were determined by PCR-restriction fragment length polymorphism (RFLP).

## MATERIALS AND METHODS

### Subjects

All the cases of this study were patients with stroke admitted to the Neurology Department of Nanjing Brain Hospital from 2009 to 2010, they were all Han Chinese, and were classified into four groups: 52 patients with LAA and 85 patients with SAO according to the TOAST typing, 50 patients with ICH, and 66 healthy people as control. The inclusion criteria were as followes: all the subjects of case groups were 50 to 80 years old, either male or female, with new-onset or recurrent stroke. The diagnoses were based on clinical history, physical examination and CT or MRI imaging. The subjects of the control group were healthy people of the same age range, without stroke history or abnormality of MRI imaging, either male or female. The exclusion criteria for the LAA and SAO group were as follows: patients with cardiac thrombotic cerebral infarction, watershed cerebral infarction, cerebral infarction caused by infective or immunological arteritis, atrial fibrillation, severe hepatic or nephritic dysfunction, cancer, autoimmune disease and hypercoagulability caused by hematological disease or drugs should be excluded; for the ICH group, patients with subarachnoid hemorrhage, traumatic intracranial hemorrhage, intracranial hemorrhage after infarction, severe hepatic or nephritic dysfunction, cancer, autoimmune disease and coagulation dysfunction caused by hematological disease or drugs should be excluded. The following data of all the subjects were recorded: gender, age, height and weight, body mass index (BMI), history of smoking, alcohol, hypertension, diabetes and heart disease, the levels of triglyeride (TG), cholesterol (CHO), high density lipoprotein (HDL), low density lipoprotein (LDL), apolipoprotein A (apoA), apolipoprotein B (apoB), and lipoprotein (a) [Lp(a)] (***Table 1***). All the study participants provided informed consents.

**Table 1:** Demographic characteristics of the subjects

Variable	LAA *(n* = 52)	SAO *(n* = 85)	ICH *(n* = 50)	Control *(n* = 66)	χ^2^/F	*P*
Women (%)	34.6	40.0	32.0	47.0	3.245	0.355
Age (y)	64.0±8.2*	66.8±7.6*	62.8±7.8*	58.7±6.1	14.520	<0.001
BMI (kg/m^2^)	24.4±4.1	24.6±4.5	24.3±4.1	23.89±3.3	0.290	0.833
Smoking (%)	30.8	22.4	18.0	18.2	3.680	0.298
Drinking (%)	11.5	10.6	18.0	7.6	3.201	0.362
Hypertension (%)	65.4*	59.3*	64.0*	25.8	30.630	<0.001
Diabetes (%)	34.6*	27.1*	6.0	6.1	27.490	<0.001
Heart disease (%)	9.6*	7.1*	4.0*	0	9.638	0.022
TG (mmol/L)	1.69±0.88	1.49±0.94	1.51±0.78	1.56±1.04	0.460	0.708
CHO (mmol/L)	4.71±1.15	4.51±1.13	4.60±0.82	4.71 ± 0.80	0.590	0.620
HDL (mmol/L)	0.99±0.31*	1.06±0.30	1.07±0.30	1.16±0.34	2.610	0.052
LDL (mmol/L)	2.70±0.86	2.60±0.92	2.51±0.65	2.59±0.64	0.420	0.739
ApoA (mmol/L)	0.98±0.24*	1.03 ± 0.21*	1.06±0.23	1.13±0.26	3.600	0.013
ApoB (mmol/L)	0.98±0.26	0.89±0.21	0.92±0.17	0.93±0.14	2.090	0.103
Lp(a) (mmol/L)	349.21 ± 391.23*	261.09±269.57	283.79±297.60	203.91±163.46	1.960	0.121

*Compared with the control group, *P* < 0.05. ApoA: apolipoprotein A; ApoB: apolipoprotein B; BMI:body mass index; CHO:cholesterol; HDL:high density lipoprotein; ICH: intracranial hemorrhage stroke; LAA: large-artery atherosclerotic stroke; LDL: low density lipoprotein; Lp(a): lipoprotein(a); SAO: small-artery occlusion lacunar stroke; TG: triglyceride.

### SNP selection and genotyping

**Fig. 1:**
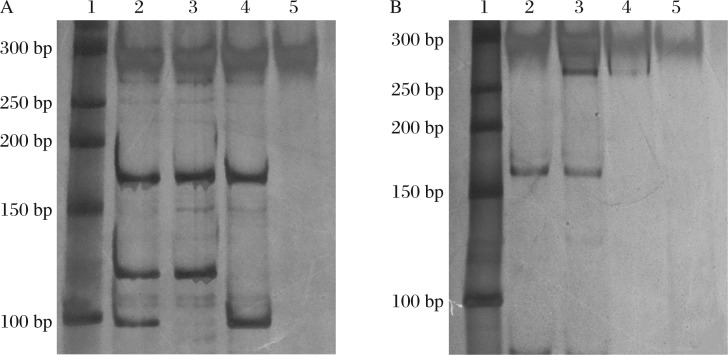
Electrophoretogram of the typical PCR products of *rs12425791* (A) and *rs11833579* (B) after digestion. A: Lane 1 is molecular weight marker, Lane 2, 3, 4 is the *AG, AA, GG* genotype, respectively, and Lane 5 is the blank control (17 bp are not visible). B: Lane 1 is molecular weight marker, Lane 2, 3 is the *AA, AG* genotype, respectively, Lane 4 is the *GG* genotype (lighter than others ), and Lane 5 is the blank control.

Five mL venous blood samples were drawn from all the subjects after fasting for at least 8 h. Genomic DNA was extracted (TIANamp, Tiangen).The primers were synthesized by Sangong Bioengineer Ltd, Shanghai. The sequences of primers for *rs12425791* were: forward 5′-GGCGAGCTGCTGCTTTTAG-3′, reverse 5′-TGTCAGAGGAGAAACCAGGAAC-3′; for *rs11833579*: forward 5′-AGGTGGGAGGATTGCTTG-3′, reverse 5′-TTTCCCTCTATTCAGCCAGAT-3′. The premix 2×*Taq* PCRmastermix (Bioedify, Nanjing, China) was used in the PCR assay. The assay mix of rs12425791 contained in a volume of 50 µL, 25 µL 2×*Taq* PCRmastermix, 5 µL genomic DNA and 0.25 µL (100 µmol/L) each primer; the thermal cycling conditions were as follows: an initial denaturation at 94°C for 5 min, 35 cycles of 94°C for 30 s, 55°C for 30 s and 72°C for 30 s, and a final extension at 72°C for 5 min. The assay mix of rs11833579 contained in a volume of 50 µL, 25 µL 2×*Taq* PCRmastermix, 3 µL genomic DNA and 0.10 µL (100 µmol/L) each primer; the thermal cycling conditions were as follows: an initial denaturation at 94°C for 5 min, 35 cycles of 94°C for 30 s, 58°C for 30 s and 72°C for 30 s, and a final extension at 72°C for 5 min. The amplification products were digested by endonucleases. The enzymes were commercially supplied by Fermentas (Canada). The amplification product of *rs12425791* were subjected to *Bsp*L I digestion, allowing differentiation of the *GG* genotype (164, 97 and 17 bp), AA genotype (164 and 115 bp) and *AG* genotype (164, 115, 97 and 17 bp). The amplification product of *rs12425791* was subjected to *Bfu* I digestion, allowing differentiation of the *GG* genotype (256 bp), *AA* genotype (169 and 87 bp) and *AG* genotype (256, 169 and 87 bp) (***Fig. 1***). The digested products were separated by PAGE and visualized by silver staining[2].

### Statistical analysis

The background data of the subjects were compared using χ^2^-test or variance analysis. Hardy-Weinberg equilibrium was performed using goodness-of-fit χ^2^-test. The allele and genotype frequencies between the case and control groups were compared using χ^2^-test or Fisher exact test. Multinomial logistic regression model was used to calculate the initial odds ratio (*OR*) of risk factors and genotypes, and 95% confidential intervals (*95%CI*) were given; if the *P*-value of some initial *OR* was less than 0.25, the corresponding risk factors or genotypes would be subjected to the model until all the *P* values < 0.05, and the adjusted *OR* and *95%CI* were given. A value of *P* < 0.05 was considered as significant. All the statistical analyses were performed using the Stata10.0 package.

## RESULTS

The genotype counts were in Hardy-Weinberg equilibrium in both the control and case groups at the two polymorphic loci (*P* = 0.15 for the control group, 0.13 for the LAA group, 0.20 for the SAO group, 0.85 for the ICH group).The genotype frequencies and allele frequencies of the two SNPs in the different groups were shown in ***Table 2***. The genotype frequencies of *rs12425791* in the LAA group and the SAO group were significantly different from that of the control group, whereas these differences were not found in the ICH group. The allele frequencies of *rs12425791* in the SAO group were significantly different from those of the control group. In regard to *rs11833579*, there was not any significant difference in genotype or allele frequencies.

Multinomial logistic regression was used to calculate the *OR* values of the genotypes of the two SNPs, and the results are shown in ***Table 3***. Before other risk factors were adjusted, the *AG* and *AA*+*AG* genotype in the LAA group and SAO group were significantly associated with stroke, while this was not found in the ICH group. After adjustment of other risk factors such as age, smoking, hypertension, diabetes, TG, CHO, LDL, HDL, apoA, apoB and Lp(a) the *AG* genotype of the LAA group and the *AG* and *AA*+*AG* genotypes of the SAO group were still significantly associated with stroke, while there was not significant association between the *AA*+*AG* genotype and stroke in the LAA group after adjustment of other risk factors mentioned above, the *AG* and *AA*+*AG* genotypes of the ICH group were not significantly associated with stroke even after adjustment other risk factors mentioned as above. All genotypes of *rs12425791* were not associated with stroke, no matter before or after adjustment. As the samples of the *AA* genotype of the two SNP sites in all groups were relatively few, the *AA* genotype was not subjected to multinomial logistic regression analysis.

**Table 2:** The genotypes and allele frequencies in each group [*n*(%)]

Group	Genotype				Allele	
	GG	AG	AA	AA±AG	G	A
*rs12425791*						
Control	49(74.2)	14(21.2)	3(4.5)	17(25.8)	112(84.8)	20(15.2)
LAA	28(53.8)	23(44.2)*	1(1.9)	24(46.1)*	79(76.0)	25(24.0)
SAO	46(54.1)	36(42.4)*	3(3.5)	39(45.9)*	128(75.3)	42(24.7)*
ICH	33(66.0)	15(30.0)	2(4.0)	17(34.0)	81(81.0)	19(19.0)
*rs11833579*						
Control	35(53.0)	24(36.4)	7(10.6)	31(47.0)	94(71.2)	38(28.8)
LAA	20(38.5)	25(48.1)	7(13.5)	32(61.5)	65(62.5)	39(37.5)
SAO	36(42.4)	38(44.7)	11(12.9)	49(57.6)	110(64.7)	60(35.3)
ICH	24(48.0)	19(38.0)	7(14.0)	26(52.0)	67(67.0)	33(33.0)

*Compared with the control group, *P* < 0.05. LAA: large-artery atherosclerosis; SAO: small-artery occlusion lacunar; ICH: intracerebral hemorrhage.

**Table 3:** Initial and adjusted *OR* values of genotypes in each case group

Group		GG	AG	AA±AG
*rs12425791*				
LAA	Initial *OR* (*95% CI*)	1.000	2.875*(1.278-6.466)	2.471*(1.137-5.366)
	Corrected *OR* (*95% CI*)	1.000	4.298*(1.430-12.922)	2.679 (0.944-7.598)
SAO	Initial *OR* (*95% CI*)	1.000	2.739*(1.311-5.723)	2.444*(1.217-4.908)
	Corrected *OR* (*95% CI*)	1.000	3.923*(1.417-10.860)	2.937*(1.119-7.710)
ICH	Initial *OR* (*95% CI*)	1.000	1.591 (0.679-3.728)	1.485 (0.664-3.319)
	Corrected *OR* (*95% CI*)	1.000	2.296 (0.773-6.818)	1.747 (0.613-4.982)
*rs11833579*				
LAA	Initial *OR* (*95% CI*)	1.000	1.823 (0.832-3.995)	1.806 (0.863-3.782)
	Corrected *OR* (*95% CI*)	1.000	1.848 (0.670-5.099)	1.814 (0.701-4.692)
SAO	Initial *OR* (*95% CI*)	1.000	1.539 (0.771-3.072)	1.537 (0.805-2.935)
	Corrected *OR* (*95% CI*)	1.000	1.551 (0.626-3.844)	1.576 (0.671-3.706)
ICH	Initial *OR* (*95% CI*)	1.000	1.155 (0.521-2.557)	1.223 (0.586-2.553)
	Corrected *OR* (*95% CI*)	1.000	0.968 (0.355-2.643)	1.180 (0.468-2.979)

*Compared with the GG group, *P* < 0.05. LAA: large-artery atherosclerosis; SAO: small-artery occlusion lacunar; ICH: intracerebral hemorrhage.

## DISCUSSION

The *NINJ* gene was first found in the study on the gene expression of Schwann cell during peripheral nerve regeneration, and then its homologue was subsequently found[3],[4]. Thus, the original *NINJ* was renamed the *NINJ1* genes, and its homologues was named the *NINJ2* gene. The molecules coded by the two genes are membrane protein, which could mediate cell adhesion[4],[5]. Ninjurin plays an important role in the peripheral nerve injury and regeneration process[5]. The two molecules can also promote axonal growth[5]. So far, no studies have shown an association between the *NINJ2* gene with stroke. The two SNPs in this study are close to the 5′ end of the *NINJ2* gene, indicating that *NINJ2* may play an important role in stroke.

Besides stroke, the *NINJ* gene was believed to play an important role in many other neurological diseases, such as multiple sclerosis, Alzheimer's disease, leprosy, hereditary sensory neuropathy and Charcot-Marie-Tooth disease[6]–[11]. Possible mechanisms included homologue assembly, Wnt signaling pathway, NF-κB signaling pathway and P53 signaling pathway[12]–[17].

Our study showed that the minor allele (*A*) of SNP *rs12425791* in the Chinese Han population would increase the susceptibility to ischemic stroke. This was the same as the conclusion from other studies on other racial groups[1], indicating that the association between SNP *rs12425791* and ischemic stroke might be general in different races. This would help us further explore the underlying mechanism of this association. However, we failed to observe any association between SNP *rs11833579* and stroke, which was contradictory to previous report[1]. We presumed that the association between SNP rs11833579 and ischemic stroke may be related to racial differences, because a previous study also failed to find an association between SNP *rs11833579* and stroke in the Black population[1].

The sample size of this study was relatively small, and it is a case-control study limiting the conclusion of the study. The study provided the preliminary data on the genotype distribution of the two SNP loci in Chinese Han population and the relationship between them and stroke. Prospective studies with a larger sample population are further being explored.

In conclusion, the SNP *rs12425791* is significantly associated with ischemic stroke in Chinese Han population, and the *A* allele increases the risk of susceptibility to stroke. The SNP *rs11833579* is not significantly associated with stroke in Chinese Han population.
